# Accessory V^6^ during thoracoscopic middle lobectomy: “an uncomfortable presence”

**DOI:** 10.1002/rcr2.601

**Published:** 2020-06-16

**Authors:** Dario Amore, Roberto Scaramuzzi, Dino Casazza, Pasquale Imitazione, Emanuele Muto, Roberta Lieto

**Affiliations:** ^1^ Department of Thoracic Surgery Monaldi Hospital Naples Italy; ^2^ Department of Respiratory Diseases Monaldi Hospital Naples Italy; ^3^ Department of Diagnostic Imaging, General Radiology Monaldi Hospital Naples Italy

**Keywords:** Lung cancer, lung injury, thoracic surgery

## Abstract

The identification of the accessory vein draining the superior segment of the right lower lobe (accessory V^6^), during the posterior mediastinal lymph node dissection, can help avoid operative complications.

## Graphical abstract

We report a patient in whom a supernumerary venous branch draining the superior segment of the right lower lobe into the left atrium (accessory V6) was accidentally discovered near the level 7 nodal packet thus avoiding complications during surgery. The anomalous vessel was retrospectively identified on chest computed tomography (CT) scan.

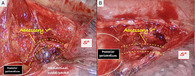

## Clinical Image

**Figure 1:**
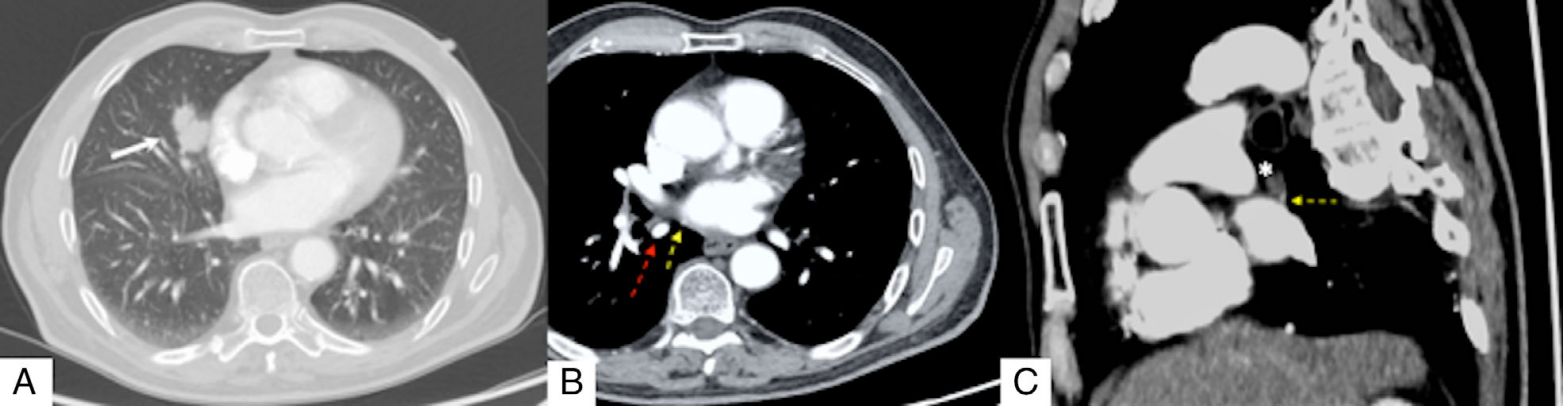
Chest computed tomography (CT) scan. (A) Lung cancer located in the middle lobe (white arrow). (B) Mediastinal window image shows vein (red arrow) and accessory vein (accessory V^6^) (yellow arrow) draining the superior segment of the right lower lobe. (C) Sagittal multiplanar reconstructed (MPR) projection shows accessory V^6^ (yellow arrow) adjacent to mediastinal lymphadenopathy (white asterisk).

**Figure 2:**
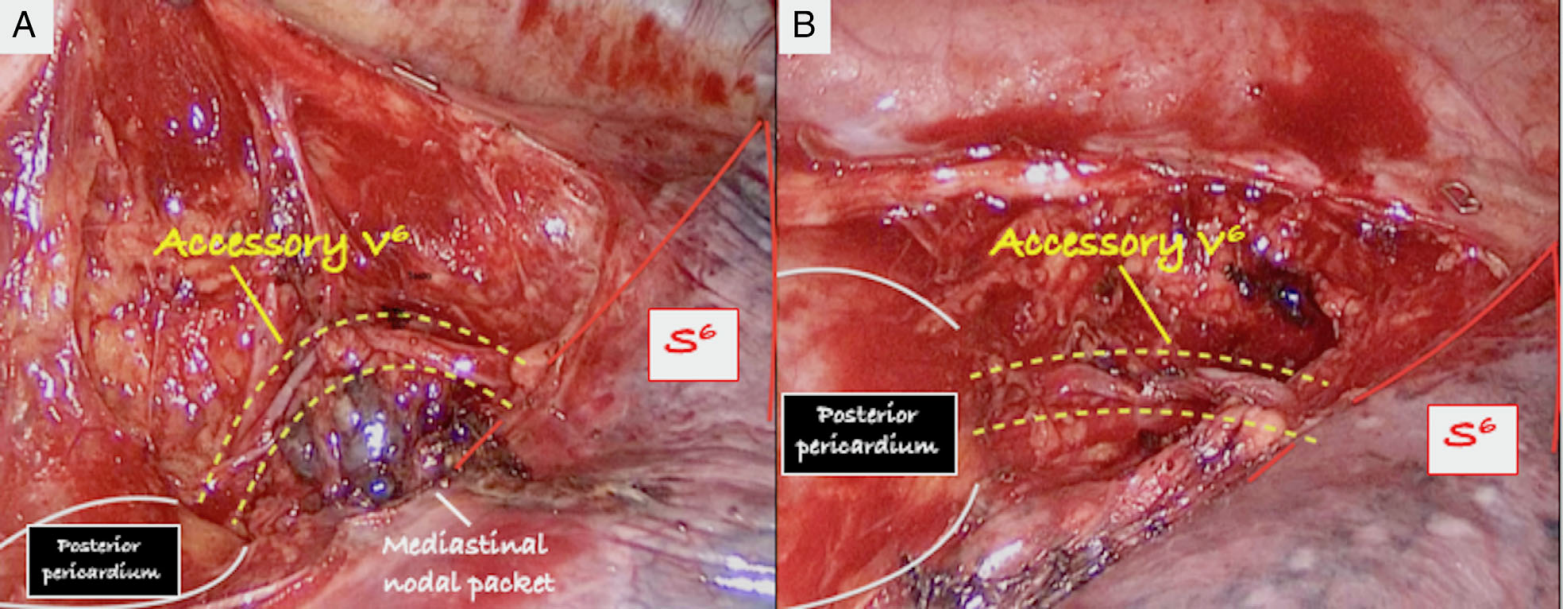
Intraoperative view during mediastinal lymph node dissection. (A) Accessory vein (accessory V^6^) draining the superior segment of the right lower lobe (S^6^) into the left atrium, displaced by posterior mediastinal lymph nodes. (B) Course of the accessory V^6^ along the posterior mediastinum after lymph node exeresis.

A 78‐year‐old man was admitted to our unit for surgical treatment of non‐small cell lung cancer (NSCLC) arising from the middle lobe (Fig. 1A). A thoracoscopic middle lobectomy was performed by a standardized three‐port anterior approach. During the surgical procedure, the posterior mediastinal pleura was divided and a supernumerary venous branch draining the superior segment of the right lower lobe into the left atrium (accessory V^6^) was accidentally discovered near the level 7 nodal packet (Fig. 2A). The vein was preserved (Fig. 2B) and required a careful mediastinal lymph node dissection to avoid annoying operative bleeding due to vessel injury and blood flow from the left atrium. The anomalous vessel was retrospectively identified on chest computed tomography (CT) scan (Fig. 1B, C).

### Disclosure Statement

Appropriate written informed consent was obtained for publication of this case report and accompanying images.

